# Takotsubo syndrome in patients with myasthenia gravis: a systematic review of previously reported cases

**DOI:** 10.1186/s12883-019-1523-z

**Published:** 2019-11-12

**Authors:** Devarajan Rathish, Minuri Karalliyadda

**Affiliations:** Department of Pharmacology, Faculty of Medicine and Allied Sciences, Rajarata University of Sri Lanka, Saliyapura, Anuradhapura, Sri Lanka

**Keywords:** Broken heart syndrome, Stress cardiomyopathy, Apical ballooning, Transient left ventricular dysfunction, Ampulla cardiomyopathy, Gebrochenes-Herz syndrome, Myasthenic crisis

## Abstract

**Background:**

Myasthenia gravis associated takotsubo syndrome is a rare condition. This study aimed to explore its typical presentation, investigations and treatment through a systematic review of previously reported cases.

**Methods:**

Databases and reference lists of the selected articles were searched for case reports on Myasthenia gravis associated takotsubo syndrome. CARE guidelines were used for the quality assessment of the selected articles.

**Results:**

Sixteen cases were selected out of 580 search results. Western Pacific, American and European regions contributed to 88% of the cases. Females were most affected (81%). Features of both myasthenia gravis and takotsubo syndrome were the common clinical presentations. All cases had a myasthenic crisis. Half of the cases had no prior diagnosis of myasthenia gravis. Pyridostigmine and prednisolone were useful for myasthenia gravis while dobutamine was most commonly used for takotsubo syndrome. All cases survived except four (25%).

**Conclusions:**

Myasthenia gravis associated takotsubo syndrome via a myasthenic crisis is rare but life-threatening. Therefore, predisposition due to emotional and physical triggers needs to be avoided for its prevention. The rare entity should be suspected even in patients without a prior diagnosis of Myasthenia gravis.

## Background

Myasthenia gravis (MG) is an autoimmune disorder caused by autoantibodies against the postsynaptic membrane at the neuromuscular junction, mostly against acetylcholine receptors (AChR) [1] and in some against muscle-specific receptor tyrosine kinase (MuSK) [2, 3]. Also, autoantibodies against low-density lipoprotein receptor-related protein (LRP4) were found in AChR and MuSK antibody-negative MG patients [4]. MG means *“grave, or serious, muscle weakness”* [5] and it causes fatigable weakness of skeletal muscles (ocular, facial, oropharyngeal, limb and respiratory) [1]. Classification of MG subgroups is as follows: early-onset MG (AChR), late-onset MG (AChR), thymoma MG (AChR), MuSK MG, LRP4 MG, seronegative MG (no autoantibodies detected) and ocular MG (AChR, MuSK, LRP4 or none). Myasthenic crisis is a life-threatening, often spontaneous event in MG which is associated with respiratory failure [6] and a hospital mortality rate of 4.5% [7]. Patients may need intubation and mechanical ventilation depending on the clinical status [8]. Rarely, myasthenic crisis is the initial presentation of MG. The crisis may be triggered by stressors like infection, surgery, pregnancy, childbirth or drugs [8, 9]. Plasma exchange and intravenous immunoglobulin are used in the management of myasthenic crisis [10]. Incidence, prevalence and the mortality rate for MG were 1.7–21.3, 15–179 and 0.06–0.89 per million person-years [11]. Incidence increased with age in both sexes. The highest incidence was observed between 60 and 80 years. Male predominance was observed among older age group while female sex showed a bimodal distribution [11]. MG is a multisystem disorder with cardiac involvement estimated to occur in 16% of cases [12]. Arrhythmia [13], pericarditis [14] and myocarditis [15] have been reported to occur in association with MG. Rarely MG could lead to Takotsubo syndrome (TTS).

TTS is “*characterised by transient systolic dysfunction of the apical and/or mid-segments of the left ventricle that mimics acute myocardial infarction but with no obstructive coronary artery disease”* [16–19]. An echocardiogram or a left ventriculogram of the patient with TTS appears like a Japanese octopus fishing pot (*Takotsubo*) [20]. The disease is also known as takotsubo cardiomyopathy, broken heart syndrome, stress cardiomyopathy, stress-induced cardiomyopathy, apical ballooning, apical ballooning cardiomyopathy, transient left ventricular dysfunction, reversible left ventricular dysfunction, ampulla cardiomyopathy and Gebrochenes-Herz-syndrome. The prevalence of TTS was 2% in all patients presenting with the acute coronary syndrome [21]. The vast majority of the patients with TTS were found to be aged ≥50 years and were females [21]. Emotional and physical stressors can lead to TTS [21].

Pathogenesis of the TTS is attributed to adrenergic overstimulation by systemic catecholamine surge which can cause acute coronary and peripheral vasospasm followed by peripheral vasodilation and left ventricular systolic dysfunction [21]. Patients with MG could have a myasthenic crisis which could subsequently trigger a TTS. The objective was to synthesise knowledge related to the rare clinical condition of MG associated TTS. We aim to systematically review globally reported cases of MG associated TTS concerning its presentation, investigations and treatment. In 2018, 2016 and 2014 a total of five separate summarises on MG associated TTS patients were published as part of a case report [22–25] and as a correspondence [26]. However, the present systemic review includes additional cases and in-depth analysis.

## Methods

### Eligibility criteria

All published case reports on MG associated TTS were included. The diagnosis of MG and TTS for eligibility was done by using the reporting physician’s clinical diagnosis. Standard diagnostic criteria were not used to confirm the diagnosis because it was impossible to apply it retrospectively based purely on reported data. Reports in non-English language were excluded. Reports were not excluded based on the year of publication or patient population.

### Information sources and search strategy

**Fig. 1:**
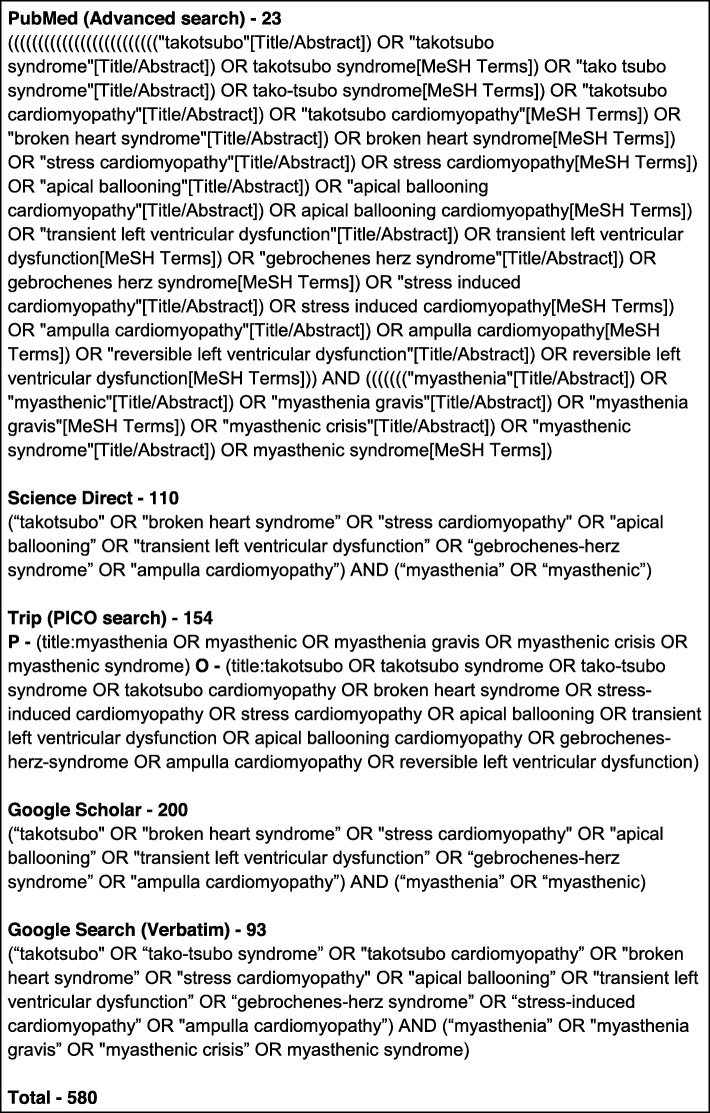
Keywords for databases and the number of search results

The search was done from early inception to September 2018. Electronic databases were searched using strings of keywords (Fig. 1). Following databases were used for the search: PubMed (Advanced search) [27], Science Direct (Advanced search) [28], Trip (PICO search) [29] and Google Scholar (Advanced search) [30]. Also, grey literature was done using Google Search (Verbatim). Further, the reference lists of the selected studies were checked for relevant articles. MeSH and other relevant terms related to each search engine were used to obtain optimum data.

### Study selection

DR and MK were involved in study selection independently. DR performed a comprehensive literature search. MK independently screened the titles and abstracts of all identified studies for selection, according to the inclusion criteria. The selected study was independently reviewed by DR to confirm the eligibility. DR and MK independently extracted data and the datasheet was finalized by consensus.

### Data collection process, data items and data analysis

Demographic data, clinical presentation, investigation findings and the management plan were extracted from the selected studies. The proposed diagnostic criteria for MG [31] and the international Takotsubo diagnostic criteria (InterTAK Diagnostic Criteria) [32] were used to summarize the findings. However, the above two criteria were not considered to determine the eligibility of case reports for selection. SI units were used to present the units of measurements. The data were analysed using Microsoft Excel (Additional file 1). Descriptive statistics were used to describe the data. The quality of the selected reports was assessed using the CARE guidelines [33]. One point was given for each item of the CARE guidelines, i.e. a maximum score of 30 for a report. The review was reported according to the Preferred Reporting Items for Systematic survey and Meta-Analysis (PRISMA) statement [34] (Additional file 2).

## Results

### Selected case reports

**Fig. 2:**
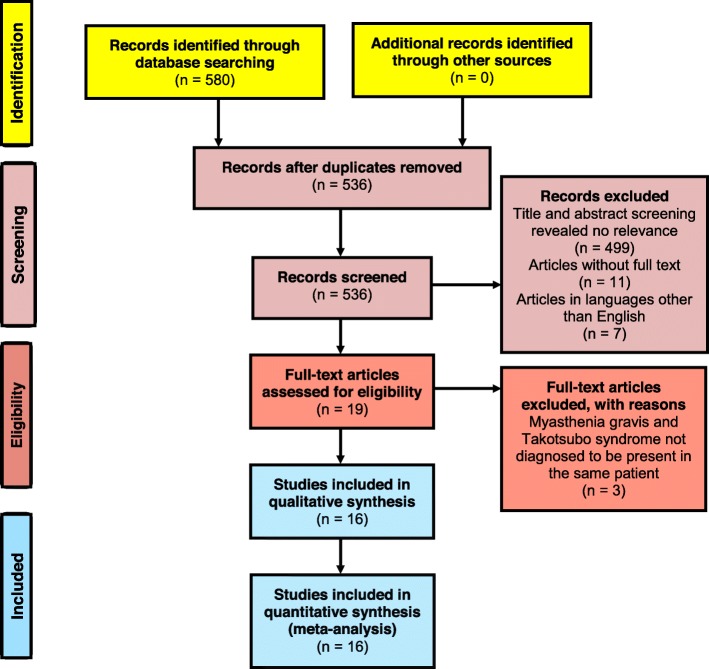
Flow diagram showing the selection process of articles for this review, according to PRISMA 2009

A total of 580 results were found from the databases (Fig. 2). After removal of duplicates, 536 articles were included for the title and abstract screening. Out of which 499 articles were excluded due to irrelevance to the study objective; 11 articles were excluded due to unavailability of full-text and another 07 articles were excluded as they were in Chinese [35], French [36], Indonesian [37], Japanese [38, 39] and Spanish [40, 41] languages. The full-texts of the remaining 19 articles were examined, and 03 were excluded as MG and TTS were not diagnosed to be present in the same patient [42–44]. Following the above screening steps, 16 articles [22–25, 45–56] were selected for the review (Fig. 2). According to the quality assessment, the mean score achieved by the selected articles was 20.4 (SD ± 2.3). The maximum score was 24 out of 30, and the minimum score was 16. Summary of scores for each item of the CARE checklist is given in Additional file 3.

### Demographic data

Additional file 1 contains data from each of the 16 reports selected for this review. Seven case reports were from the region of Americas (44%), four from Western Pacific (25%), three from Europe (19%) and two from South-East Asia (13%). There were no case reports from the African or Eastern Mediterranean regions. Country wise, United States of America topped the list with four reported cases followed by India and Japan having two cases each. All other countries (Austria, Australia, Brazil, Colombia, Italy, Mexico, Singapore and the United Kingdom) reported one case each. The mean age of the patients was 61.6 (SD 14.1) years, with an age range of 34–80 years. There were 13 (81%) female patients. According to this review, the first case reported on MG associated TTS was by Sousa JMA et al. in 2005 from Brazil [50]. The latest report was from Austria by Finsterer J et al. in 2018 [22].

### Clinical presentation

Half of the selected cases presented with muscle weakness while shortness of breath was the second most common presentation (44% - 7/16) followed by dysphagia in 25% (4/16). System-wise summary of the clinical presentations is shown in Table 1 and Additional file 1.

**Table 1:** Presenting complaints of patients who presented with both Myasthenia gravis and Takotsubo syndrome

Item	Cases reported out of 16 (%)
General	
Muscle weakness	8 (50)
Chest pain	6 (38)
Fatigue	3 (19)
Myalgia	2 (13)
Fever	1 (6)
Sweating	1 (6)
Respiratory	
Shortness of breath	7 (44)
Cough	3 (19)
Aspiration	1 (6)
Dyspnea	1 (6)
Respiratory failure	1 (6)
Rhinorrhoea	1 (6)
Wheezing	1 (6)
Cardiovascular system	
Palpitation	1 (6)
Nervous system	
Diplopia	2 (13)
Dysarthria	2 (13)
Slurred speech	2 (13)
Headache	1 (6)
Gastro-intestinal system	
Dysphagia	4 (25)
Difficulty in mastication	2 (13)
Difficulty in swallowing	2 (13)
Nasal regurgitation	1 (6)
Nausea	1 (6)

### Summary of results for myasthenia gravis

Symptoms of respiratory disorder (81%), blepharoptosis (63%), dysphagia (56%) and limb muscle weakness (50%) were observed in more than half the cases. Anti-acetylcholine receptor (AChR) antibody was positive in 50% (8/16). Among the tests for neuromuscular junction disorders, edrophonium chloride (Tensilon) test was positive in 25% (4/16) (Table 2). The myasthenic crisis was experienced by all patients. Thymoma was present in only 25% of the cases. Three cases had results for strational muscle antibody out of which only one had elevated levels.

**Table 2:** Summary of results for the diagnostic criteria on Myasthenia gravis [31]

Item	Frequency out of 16 (%)
A. Symptoms	
Blepharoptosis	10 (63)
Eye movement disorder (Diplopia - 6, Opthalmoparesis - 2)	6 (38)
Facial muscle weakness (Facial diplegia - 4)	4 (25)
Dysarthria	6 (38)
Dysphagia	9 (56)
Mastication disorder	5 (31)
Cervical muscle weakness	4 (25)
Limb muscle weakness	8 (50)
Respiratory disorder	13 (81)
B. Pathogenic autoantibodies	
Positive antiacetylcholine receptor (AChR) antibody	8 (50)
C. Neuromuscular junction disorders	
Positive on eyelid easy fatigability test	2 (13)
Positive on edrophonium chloride (Tensilon) test	4 (25)
Positive on repetitive stimulation test	3 (19)

### Summary of results for Takotsubo syndrome

The most common type of primary TTS was classical (75%) followed by global (13%), basal (6%) and midventricular (6%) types. Recurrence of TTS was observed in two cases, and both were classical type of TTS. Left ventricular dysfunction (100%), elevated troponin level (75%) and T-wave inversion (50%) were observed in more than half the reported cases (Table 3). Myasthenic crisis could be implicated as a possible trigger for TTS in all cases. In addition to the electrocardiography (ECG) changes mentioned in the international Takotsubo diagnostic criteria, the following changes were also observed in one case each: left axis deviation, narrow complex supraventricular tachycardia, occasional ventricular premature contractions, right bundle branch block, Torsades de Pointes and tachyarrhythmia (Additional file 1). Atrial fibrillation was found in 3 cases. According to the diagnostic criteria, exclusion of infectious myocarditis via a cardiac magnetic resonance imaging was not reported in any of the selected cases. The diagnostic criteria state that post-menopausal women are more prone to experience a TTS. However, data were insufficient to find if the female cases were in their post-menopausal period. Additional cardiovascular, respiratory and neurological examination findings are summarised in Additional file 1.

**Table 3:** Summary of results for the diagnostic criteria on Takotsubo syndrome [32]

Item	Frequency out of 16 (%)
1. Left ventricular dysfunction (hypokinesia - 8, akinesia - 7, dyskinesia - 1)	16 (100)
2. Presence of a trigger (emotional, physical, or combined)	16 (100)
3. Neurologic disorders (e.g. subarachnoid haemorrhage, stroke/transient ischaemic attack, seizures or pheochromocytoma)	0 (00)
4. New Electrocardiography abnormalities	
ST-segment elevation	7 (44)
ST-segment depression	1 (6)
T-wave inversion	8 (50)
QTc prolongation	2 (13)
5. Levels of cardiac biomarkers	
Elevated troponin level	12 (75)
Elevated creatine kinase level (CK - 3, CKMB - 3, both - 1)	7 (44)
6. Coronary artery disease	4 (25)

### Past medical history

Past medical history of MG was found in 50% of the patients out of which 88% (7/8) was general disease and the remaining was an ocular disease. The age of onset of MG ranged from 34 to 77 years with a mean of 56.4 (SD 14.1) years. Hypertension (25%), atrial fibrillation (13%) and hypothyroidism (13%) were the next three common past medical diseases (Table 4). Majority of the patients who had a history of MG were reported to have received anti-cholinesterase medication (75% - 6/8), and pyridostigmine was received by five of them (Table 5). Other past drug history included bisoprolol, candesartan, disopyramide, escitalopram, furosemide, losartan, L-thyroxin, methimazole and periciazine in one case each.

**Table 4:** Past medical history of patients who presented with both Myasthenia gravis and Takotsubo syndrome

Item	Cases reported out of 16 (%)
Myasthenia gravis (General - 7, Ocular - 1)	8 (50)
Hypertension	4 (25)
Atrial fibrillation	2 (13)
Hypothyroidism	2 (13)
Asthma	1 (6)
Chronic obstructive pulmonary disease	1 (6)
Crohn’s disease	1 (6)
Depression	1 (6)
Diabetes mellitus	1 (6)
Grave’s ophthalmopathy	1 (6)
Heart failure	1 (6)
Hoarseness of voice due to the injured recurrent laryngeal nerve	1 (6)
Hyperthyroidism	1 (6)
Hypoferric anaemia	1 (6)
Myocardial infarction	1 (6)
Polymyalgia rheumatica	1 (6)
Thyroid adenoma	1 (6)

**Table 5:** Myasthenia gravis related drug history

Item	Cases reported out of 8^a^ (%)
Anti-cholinesterase (Pyridostigmine - 5, Neostigmine - 2, Drug unidentified - 1)	6 (75)
Corticosteroids (Prednisolone - 2, Methylprednisolone - 1)	3 (19)
Intravenous immunoglobulin	1 (6)

^a^Only 8 cases were reported to have had a history of Myasthenia gravis

### Additional investigation findings

Chest X-ray revealed hilar infiltrates in 3 cases while cardiac dilatation, enlargement of the mediastinal shadow and mild hyperinflation were observed in one case each. Further, blood gas analysis, computed tomography, coronary angiogram, left ventriculography, magnetic resonance imaging and transthoracic echocardiography were performed and the results of which are summarised in Additional file 1. Respiratory acidosis was found in 83% (5/6) and respiratory alkalosis in 17% (1/6) of the patients who had a blood gas analysis.

### Treatment modalities and survival

Among drugs for MG, more than 50% received anticholinesterase (94%) and corticosteroids (63%). Pyridostigmine (8/15) and prednisolone (8/10) were the most common anticholinesterase, and corticosteroids used respectively. Dobutamine was the most commonly used drug for TTS (31%). Prednisolone (38% - 6/16) and pyridostigmine (19% - 3/16) were used at discharge (Table 6). Intubation and ventilation were needed for 81% (13/16) of the cases for respiratory support. Plasmapheresis was performed in 50% (8/16) of cases. Only 4 (25%) patients had a thymoma, and surgical intervention was done in all four of them. Tracheostomy (3/16), pacemaker (2/16) and fluid restriction (1/19) were received by patients as part of the management.

**Table 6:** Treatment during the present admission

Item	Frequency out of 16 (%)
Drugs for Myasthenia gravis	
Anti-cholinesterase (pyridostigmine - 8, neostigmine - 5, unidentified drug - 2)	15 (94)
Corticosteroids (prednisolone - 8, hydrocortisone - 1, methylprednisolone - 1)	10 (63)
Intravenous immunoglobulin	4 (25)
Immunosuppressants (mycophenolate mofetil - 1, unidentified drug - 1)	2 (13)
Drugs for Takotsubo syndrome	
Dobutamine	5 (31)
Noradrenaline	2 (13)
Angiotensin-converting enzyme (drug unidentified)	1 (6)
Bisoprolol	1 (6)
Diuretics (drug unidentified)	1 (6)
Dopamine	1 (6)
Enalapril	1 (6)
Levosimendan	1 (6)
Ramipril	1 (6)
Treatment on discharge	
Prednisolone	6 (38)
Pyridostigmine	3 (19)
Azathioprine	1 (6)
Intravenous immunoglobulin	1 (6)
Mycophenolate mofetil	1 (6)
Neostigmine	1 (6)
Plasmapheresis	1 (6)
Radiation	1 (6)

Among the selected cases following complications were noted: respiratory failure (4/16), pulmonary oedema (2/16), respiratory arrest (2/16), acute enteritis (1/16), acute kidney injury (1/16), anemia (1/16), aspiration pneumonia (1/16), asystole (1/16), disseminated intravascular coagulation (1/16), heart failure (1/16), hypercalcemia (1/16), hypertension (1/16), hypocalcemia (1/16), hypoparathyroidism (1/16), hypoproteinemia (1/16), hypotension (1/16), hypoxia (1/16), myocardial oedema (1/16), proteinuria (1/16), renal insufficiency (1/16), sepsis (1/16), streptococcal pneumonia (1/16), supraventricular bradyarrhythmia (1/16), tonic-clonic seizure (1/16), vitamin-D deficiency (1/16). Out of the selected cases, 75% (12/16) completely recovered from their illness. Four patients died out of which two cases had a multi-organ failure secondary to heart failure and sepsis, and the cause of death was not reported in the rest of the two deaths.

## Discussion

Western Pacific, American and European regions have contributed to the vast majority (88%) of cases on MG associated TTS. It was noted that there were no cases reported from African or Eastern Mediterranean regions. Females were most affected (81%), and this is predictable as TTS is common among females [11, 21]. MG associated TTS showied a wide age range from 34 to 80 years. However, 69% (11/16) of the participants were over 60 years of age. Clinical features of both MG and TTS were commonly observed. Both MG and TTS have similar emotional and physical triggers [8, 21]. Therefore, a common trigger could have resulted in triggering them together. However, myasthenic crisis was experienced by all patients making it a possible trigger for TTS in MG. Therefore, it is necessary to be vigilant for TTS in MG patients with myasthenic crisis. Further, optimum pharmacological management in patients with MG is proposed to prevent myasthenic crisis and a subsequent TTS. Also, past medical history of MG was found only in 50% of the patients. Hence, a rare presentation of MG associated TTS could be the first presentation of MG. Further, certain cardiac agents like beta-adrenergic antagonists, calcium channel antagonists, procainamide and quinidine have the potential to trigger a myasthenic crisis [8]. Therefore, treatment of TTS using similar agents needs to be done with caution among patients with MG. The above facts highlight the importance of optimum vigilance on MG associated TTS.

The mean quality assessment score for the selected articles was 20.4 (SD ± 2.3) out of a total of 30 per article. Differences in reporting of clinical presentation, investigation findings and treatment options limited the review from producing a comprehensive summary. Also, a systematic review of case reports cannot establish a causal relationship between MG and TTS. Moreover, the lack of data on the control of other co-morbid diseases prevents us from pinpointing MG as the sole trigger of TTS. Nevertheless, the review has produced excellent information on MG associated TTS. Future prospective studies on MG associated TTS are methodologically challenging considering the unusual nature of the combined presentation. However, continuous reporting of similar cases will help improve the understanding of this rare entity.

## Conclusions

MG associated TTS is rare but can be life-threatening. Predisposition especially due to emotional and physical triggers need to be addressed for the prevention of MG associated TTS. Identification of myasthenia crisis should alert the treating clinician to look for features of TTS. Hence, the early detection could help optimise pharmacological management.

## Supplementary information

**Additional file 1.** Datasheet of the review on Takotsubo Syndrome in patients with Myasthenia Gravis, 2018. This provides the data extracted for the review.

**Additional file 2.** PRISMA 2009 checklist. This provides the PRISMA 2009 checklist related to this systematic review.

**Additional file 3.** Summary of scores for items of the CARE checklist. This provides the summary of scores for each item of the CARE checklist.

## Data Availability

All data generated or analysed during this study are included in this published article (and its additional files).

## Abbreviations

CARE: Case reports
ECG: Electrocardiography
MeSH: Medical Subject Headings
MG: Myasthenia gravis
PICO: Population, intervention, comparison and outcome
PRISMA: Preferred reporting items for systematic reviews and meta-analyses
SD: Standard deviation
TTS: Takotsubo syndrome
