# Supplementary material for: Takotsubo syndrome in patients with myasthenia gravis: a systematic review of previously reported cases
Source: BMC Neurol. 2019 Nov 12;19:281. doi: 10.1186/s12883-019-1523-z (PMC6849291; doi:10.1186/s12883-019-1523-z)
Supplement: Supplementary file 3 — Additional file 3. Summary of scores for items of the CARE checklist. This provides the summary of scores for each item of the CARE checklist. [file 12883_2019_1523_MOESM3_ESM.docx]

| **Additional file 3 - Summary of scores for items of the CARE checklist** | | |
| --- | --- | --- |
| **No** | **Item** | **Frequency out of 16 (%)** |
| 01 | The words “case report” should be in the title along with the area of focus | 1 (6) |
| 02 | 2 to 5 key words that identify areas covered in this case report | 9 (56) |
| 03 | Introduction - What is unique about this case? What does it add to the medical literature? | 10 (62) |
| 04 | The main symptoms of the patient and the important clinical findings | 6 (38) |
| 05 | The main diagnoses, therapeutics interventions, and outcomes | 11 (69) |
| 06 | Conclusion—What are the main “take-away” lessons from this case? | 12 (75) |
| 07 | One or two paragraphs summarizing why this case is unique with references | 10 (62) |
| 08 | De-identified demographic information and other patient specific information | 16 (100) |
| 09 | Main concerns and symptoms of the patient | 16 (100) |
| 10 | Medical, family, and psychosocial history including relevant genetic information (also see timeline) | 16 (100) |
| 11 | Relevant past interventions and their outcomes | 16 (100) |
| 12 | Describe the relevant physical examination (PE) and other significant clinical findings | 16 (100) |
| 13 | Important information from the patient’s history organized as a timeline | 1 (6) |
| 14 | Diagnostic methods (such as PE, laboratory testing, imaging, surveys) | 16 (100) |
| 15 | Diagnostic challenges (such as access, financial, or cultural) | 3 (19) |
| 16 | Diagnostic reasoning including other diagnoses considered | 16 (100) |
| 17 | Prognostic characteristics (such as staging in oncology) where applicable | 0 (0) |
| 18 | Types of intervention (such as pharmacologic, surgical, preventive, self-care) | 16 (100) |
| 19 | Administration of intervention (such as dosage, strength, duration) | 15 (94) |
| 20 | Changes in intervention (with rationale) | 15 (94) |
| 21 | Clinician and patient-assessed outcomes (when appropriate) | 16 (100) |
| 22 | Important follow-up diagnostic and other test results | 14 (88) |
| 23 | Intervention adherence and tolerability (How was this assessed?) | 13 (81) |
| 24 | Adverse and unanticipated events | 4 (25) |
| 25 | Discussion of the strengths and limitations in your approach to this case | 15 (94) |
| 26 | Discussion of the relevant medical literature | 15 (94) |
| 27 | The rationale for conclusions (including assessment of possible causes) | 14 (88) |
| 28 | The primary “take-away” lessons of this case report | 13 (81) |
| 29 | When appropriate the patient should share their perspective on the treatments, they received | 0 (0) |
| 30 | Did the patient give informed consent? | 3 (19) |
